# Pyridine-3-carboxamide–telluric acid (1/1)

**DOI:** 10.1107/S2056989018013579

**Published:** 2018-09-28

**Authors:** Jan Fábry

**Affiliations:** a Inst. of Physics of the Czech Academy of Sciences, Na Slovance 2, 182 21 Praha 8, Czech Republic

**Keywords:** crystal structure, hydrogen bonding, telluric acid

## Abstract

In the title structure, C_6_H_6_N_2_O·H_6_O_6_Te, the pyridine-3-carboxamide and telluric acid mol­ecules are inter­connected by conventional O—H⋯N, N—H⋯O and O—H⋯O hydrogen bonds of moderate strength as well as by π–π inter­actions between the pyridine rings. The strongest hydrogen bond in the structure is formed between a hydroxyl group of the H_6_TeO_6_ mol­ecule and the *N*-pyrimidine N atom. The structure is unusual because of presence of the alternating sheets, which contain H_6_TeO_6_ and pyridine-3-carboxamide mol­ecules, respectively. These sheets are aligned parallel to (001).

## Abstract 2

The title mol­ecules, pyridine-3-carboxamide and telluric acid, are inter­connected by conventional O—H⋯N, N—H⋯O and O—H⋯O hydrogen bonds of moderate strength as well as by π–π inter­actions between the pyridine rings.

## Chemical context   

The motivation for the title structure determination follows from the fact that there are relatively a few structure determinations of mol­ecular crystals containing the telluric acid mol­ecule H_6_TeO_6_ (Groom *et al.*, 2016 ▸). These structure determinations are summarized in Table 1 ▸.

H_6_TeO_6_ is a weak acid with p*K_a_* = 7.68 (1st degree; CRC Handbook, 2017 ▸) at room temperature. At the same time, the p*K_a_* value for pyridine-3-carboxamide is 3.3 (CRC Handbook, 2009 ▸). Δp*K_a_* = p*K_a_*(base) − p*K_a_*(acid) − 4.4, which indicates that the crystalline product would rather be a co-crystal (Childs *et al.*, 2007 ▸). In all the cases listed in Table 1 ▸, the H_6_TeO_6_ mol­ecules are fully protonated. All of these known structures are co-crystals except for ZARGII where H_6_TeO_6_ is an additive mol­ecule in the salt structure.

In most of the listed structures, the mol­ecules of H_6_TeO_6_ form columns which are inter­connected by O—H⋯O hydrogen bonds. Such a situation takes place in KUTBUW (the columns are parallel to the *a* axis), UREATE, UREATE01, UREATE02 (parallel to the *c* axis) and VALTUX (parallel to the *c* axis). Analogous columns parallel to the *a* axis are present in GUNQUB; however, these columns are formed together with water mol­ecules. In the other two structures, the constituent mol­ecules are surrounded by each other. None of the structures in Table 1 ▸ contains a hydrogen bond with disordered hydrogen atoms in which the hydroxyl groups of the telluric acid are involved. Inter­estingly, neutron diffraction experiments revealed that the cubic form of H_6_TeO_6_ (Cohen-Addad, 1971 ▸) possesses disordered hydrogen atoms, in contrast to the monoclinic form (Lindqvist & Lehmann, 1973 ▸). The H_6_TeO_6_ mol­ecule can be considered as an inter­esting building block for crystal engineering because it can offer each of its six hydroxyl groups for the formation of hydrogen bonds with neighbouring mol­ecules.

**Figure 1:**
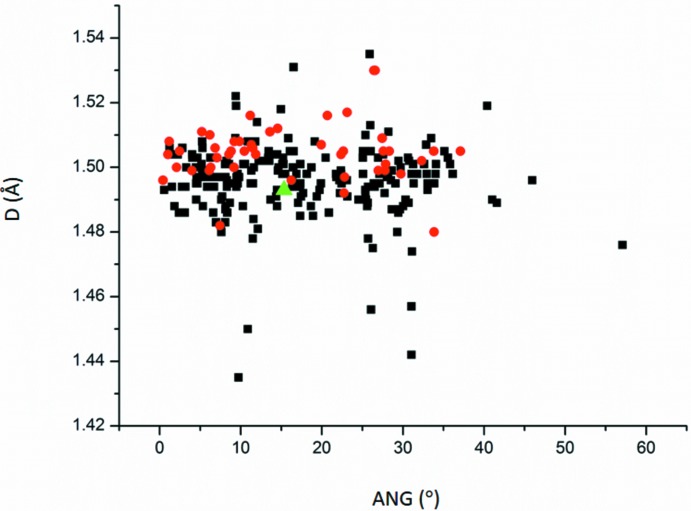
Dependence of the C—C bond distance *D*, which inter­connects the pyridine and the amide groups, on the inter­planar angle (ANG) between these groups in 3-pyridine­carboxamide mol­ecules (black squares) or 3-carbamoylpyridin-1-ium mol­ecules (red circles). The inter­planar angle has been calculated from the non-hydrogen atoms of these groups. The title structure, which belongs among 3-pyridine­carboxamide mol­ecules, is depicted by a green triangle.

3-Pyridine­carboxamide (nicotinamide) is a biologically important mol­ecule which is an active part of the vitamin B3 and nicotinamide adenine dinucleotide (NAD) [see for example Wald (1991 ▸) and Williamson *et al.* (1967 ▸)]. The inter­planar angles *ANG* between the pyridine and the amide groups in the 3-pyridine­carboxamide or 3-carbamoylpyridin-1-ium mol­ecules span a large angle because these two moieties are connected by a single C—C bond (bond *D*, Fig. 1 ▸). (This single bond corresponds to the bond C1—C6 in the title structure.) Thus 3-pyridine­carboxamide as well as 3-carbamoylpyridin-1-ium mol­ecules can easily accommodate to the environment for optimization of the amide inter­actions. It seems that the bond length *D* tends to be longer in the 3-carbamoylpyridin-1-ium mol­ecules than in 3-pyridine­carboxamide mol­ecules. This phenomenon can easily be explained by the elongation of the C—NH^+^ bonds in comparison to the the C—N bonds in the conjugated bonds system present in the pyridine rings, and thus by a tendency to a slight elongation of bond *D*.
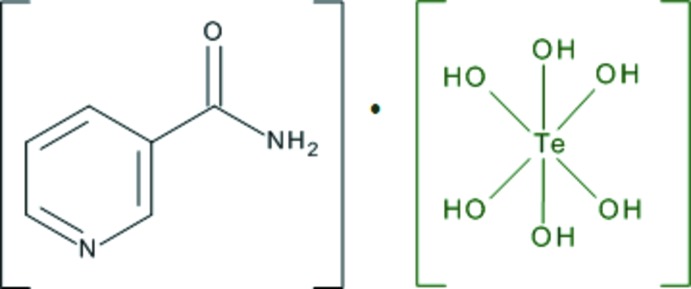


## Structural commentary   

**Figure 2:**
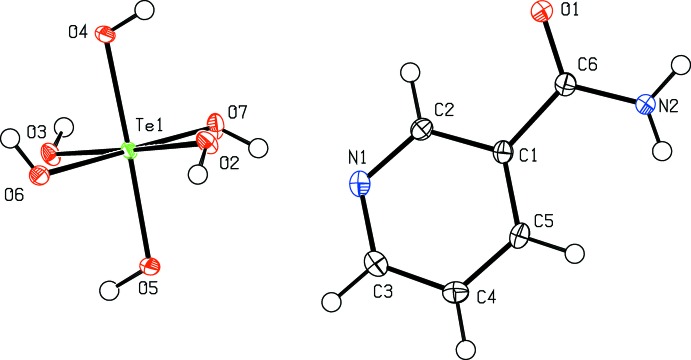
The title mol­ecule with anisotropic atomic displacements shown at the 50% probability level (*PLATON*; Spek, 2009 ▸).

The title mol­ecules are shown in Fig. 2 ▸. The inter­planar angle between the pyridine non-hydrogen atoms and the non-hydrogen amide atoms is 15.25 (8)°.

**Figure 3:**
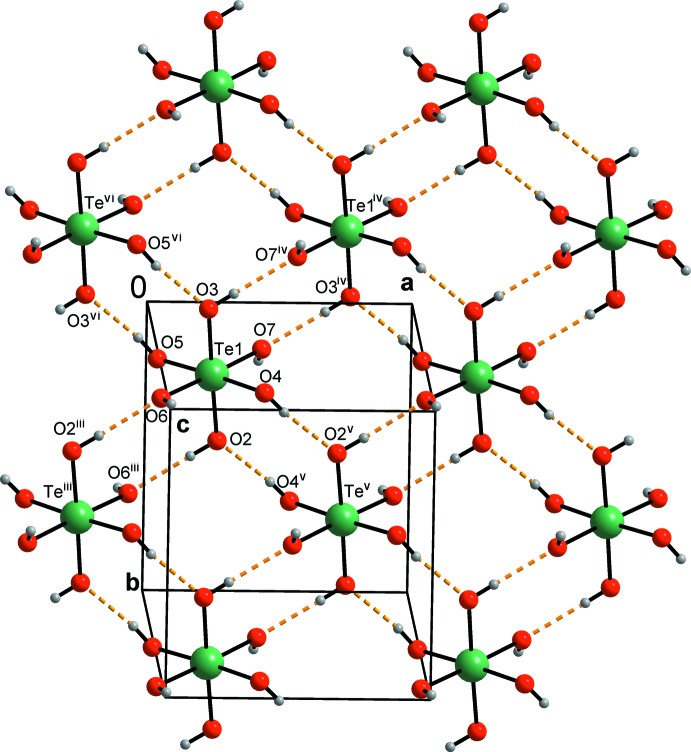
View (*DIAMOND*; Brandenburg & Putz, 2005 ▸) of a sheet composed of H_6_TeO_6_ mol­ecules only. H, O and Te atoms are represented by small grey, red and green circles, respectively. Hydrogen bonds are shown as yellow dashed lines. Symmetry codes as in Table 2 ▸.

Table 2 ▸ lists the hydrogen bonds present in the title structure. The parameters of these hydrogen bonds place them in the category of moderate hydrogen bonds (Gilli & Gilli, 2009 ▸). The sheets composed of the telluric acid mol­ecules only are held together by O—H⋯O hydrogen bonds (Fig. 3 ▸). These sheets alternate with the 3-pyridine­carboxamide mol­ecules, which are inter­connected by hydrogen bonds as well as by π-electron⋯π-electron ring inter­actions (Figs. 3 ▸–5 ▸ ▸). These sheets are parallel to (001). The presence of these sheets is so far unique among the known structures of mol­ecular crystals with H_6_TeO_6_ (see also the *Chemical context* section).

The secondary amine nitro­gen is the acceptor of the strongest hydrogen bond present in the structure (O7—H1*O*7⋯N1; Table 2 ▸). The primary amine hydrogen H1*N*2 is donated to one of the hydroxyl oxygen atoms of the telluric acid while H2*N*2 is donated to the oxygen atom of the amide group (O4).

The most important piece of knowledge derived from the study of the title structure is the functionality of the telluric acid mol­ecule, which can become a constitutional part of the hydrogen-bonding pattern. This property of the telluric acid mol­ecule has not been so far studied in depth in mol­ecular crystals because of scarcity of relevant structural data.

## Supra­molecular features   

The telluric acid mol­ecules H_6_TeO_6_ form sheets (Fig. 3 ▸) parallel to (001). Each telluric acid mol­ecule donates four hydrogen atoms to four symmetry-equivalent telluric acid mol­ecules and accepts four hydrogen atoms from these mol­ecules. These hydrogen bonds are arranged in centrosymmetric graph-set motifs 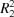
(8) (Etter *et al.*, 1990 ▸): Te1/O3/⋯H1*O*5^vi^/O5^vi^/Te1^vi^/O3^vi^⋯H1*O*5/O5; Te1/O7/⋯H1*O*3^iv^/O3^iv^/Te1^iv^/O7^iv^⋯H1*O*3/O3; Te1/O6/⋯H1*O*2^iii^/O2^iii^/Te1^iii^/O6^iii^⋯H1*O*2/O2; Te1/O2/⋯H1*O*2/O4^v^/Te1^v^/O2^v^⋯H1*O*4/O4 (symmetry codes as in Table 2 ▸).

**Figure 4:**
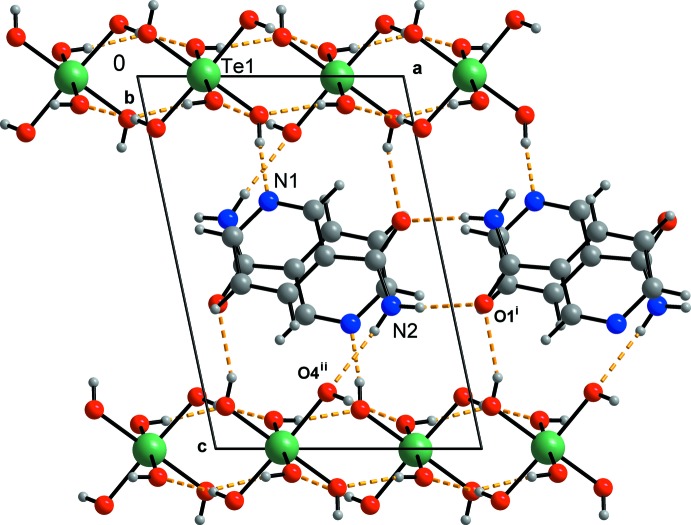
View (*DIAMOND*; Brandenburg & Putz, 2005 ▸) of the title structure along the *b* axis. C, H, N, O and Te atoms are represented by grey, small grey, blue, red and green circles, respectively. Hydrogen bonds are shown as yellow dashed lines. Symmetry codes as in Table 2 ▸

Another hydrogen atom of the telluric acid is donated to atom N1, thus forming a chain with graph-set motif *C*(3). The chain is composed of the atoms O7—H1*O*7⋯N1 (Figs. 4 ▸ and 5 ▸) and this hydrogen bond is the strongest of all the hydrogen bonds present in the title structure (Table 2 ▸).

The primary amine hydrogen atom H1*N*2 is involved in the hydrogen bond N2—H1*N*2⋯O4^ii^ (symmetry codes as in Table 2 ▸). The other primary amine hydrogen atom, H2*N*2, takes part in the centrosymmetric pair with an 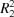
(8) graph-set motif composed of the the atoms O1/C6/N2/H2*N*2⋯O2^i^/C6^i^/N2^i^/H2*N*2^i^ (symmetry codes as in Table 2 ▸; Figs. 4 ▸ and 5 ▸).

**Figure 5:**
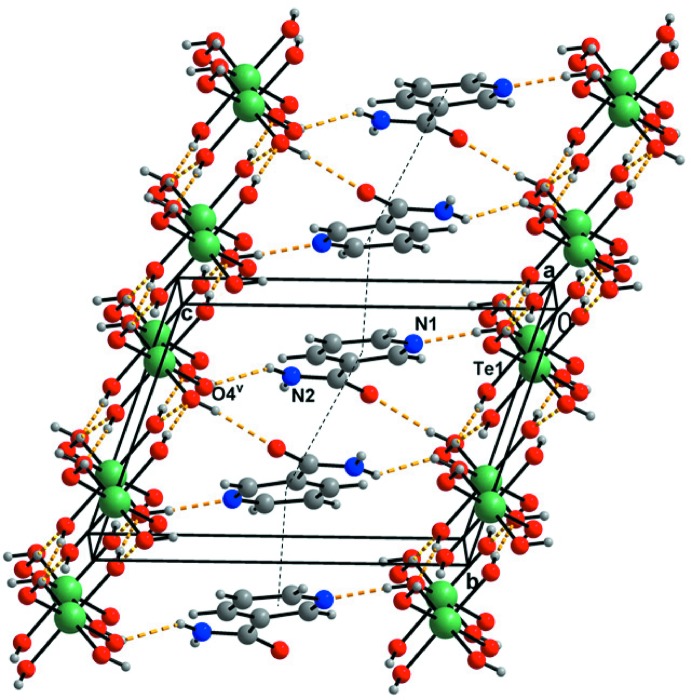
View (*DIAMOND*; Brandenburg & Putz, 2005 ▸) of the title structure along the *a* axis. C, H, N, O and Te atoms are represented by grey, small grey, blue, red and green circles, respectively. Hydrogen bonds are shown as yellow dashed lines. The π–π electron-ring interactions are indicated by black dashed lines.

The closest centroid–centroid distance [3.4101 (9) Å] indicates the presence of π–π inter­actions between adjacent pyridine rings (at *x*, *y*, *z* and −*x* + 1, −*y*, −*z* + 1) (Fig. 5 ▸).

## Synthesis and crystallization   

Equimolar amounts of 3-pyridine­carboxamide (0.40 g) and telluric acid (0.75 g) were dissolved in water (10 ml). Colourless crystals of the title compound were obtained by slow evaporation over the course of three weeks.

## Database survey   

The applied crystallographic databases were the Cambridge Crystallographic Database (version 5.39 with updates up to May 2018; Groom *et al.*, 2016 ▸) and the Inorganic Crystal Structure Database (ICSD-Web, June 2018; FIZ Karlsruhe, 2018).

## Refinement   

Crystal data, data collection and structure refinement details are summarized in Table 3 ▸. All hydrogen atoms were discernible in the difference electron-density map. The constraints C_ar­yl_—H_ar­yl_ = 0.95 Å and *U*_iso_(H_ar­yl_) = 1.2*U*_eq_(C_ar­yl_) were applied to the aryl H atoms. The positional parameters of the primary amine hydrogens H1*N*2 and H2*N*2 were refined freely, *U*_iso_(H) = 1.5*U*_eq_(N2). The positional parameters of the hydroxyl groups of H_6_TeO_6_ were refined with the distance restraints 0.84 Å with elasticities 0.02 Å (Müller, 2009 ▸); *U*_iso_(H) = 1.5*U*_eq_(O_telluric acid_). The reason for these restraints follows from quite short O—H distances, which spanned the inter­val 0.66 (2)–0.75 (2) Å if no restraint was applied. Reflection 011 was masked by the backstop and omitted from the refinement.

## Supplementary Material

Crystal structure: contains datablock(s) global, I. DOI: 10.1107/S2056989018013579/eb2012sup1.cif

Structure factors: contains datablock(s) I. DOI: 10.1107/S2056989018013579/eb2012Isup2.hkl

Supporting information file. DOI: 10.1107/S2056989018013579/eb2012Isup3.smi

Supporting information file. DOI: 10.1107/S2056989018013579/eb2012Isup4.cml

CCDC reference: 1869412

Additional supporting information:  crystallographic information; 3D view; checkCIF report

## Figures and Tables

**Table 1:** Overview of the known structure determinations of mol­ecular crystals containing the H_6_TeO_6_ mol­ecule

Refcode	Reference	Important functional groups present in the structure
BINFAF	Tran Qui *et al.* (1982 ▸)^*a*^	H_3_N^+^, COO^−^
BINFAF01	Andersen *et al.* (1983 ▸)^*a*^	H_3_N^+^, COO^−^
BINFAF02	Tran Qui *et al.* (1987 ▸)^*a*^	H_3_N^+^, COO^−^
BINFAF10	Tran Qui *et al.* (1984 ▸)^*a*^	H_3_N^+^, COO^−^
GUNQUB	Driess *et al.* (2001 ▸)^*b*^	N, NH, NH_2_
KUTBUW	Ilczyszyn *et al.* (1992 ▸)^*c*^	(H_3_C)N^+^, COO^−^
UREATE	Loub *et al.* (1979 ▸)^*d*^	NH_2_, CO
UREATE01	Loub & Dušek (1986 ▸)^*d*^	NH_2_, CO
UREATE02	Averbuch-Pouchot & Durif (1989 ▸)^*d*^	NH_2_, CO
VALTUX	Averbuch-Pouchot (1988 ▸)^*e*^	*R*_2_H_2_N^+^, COO^−^
ZARGII	Císařová *et al.* (1995 ▸)^*f*^	*R*_3_NH, COO^−^

Notes: (*a*) bis­(glycine) hexa­hydroxy­tellurium monohydrate; (*b*) bis­(adenine) hexa­hydroxy­tellurium tetra­hydrate; (*c*) bis­(betaine) telluric acid; (*d*) bis­(urea) orthotelluric acid; (*e*) sarcosine telluric acid; (*f*) disodium hexa­hydro­telluric acid di­hydrogenethyl­enedi­amine­tetra­acetate dihydrate.

**Table 2:** Hydrogen-bond geometry (Å, °)

*D*—H⋯*A*	*D*—H	H⋯*A*	*D*⋯*A*	*D*—H⋯*A*
N2—H1*N*2⋯O1^i^	0.83 (2)	2.11 (2)	2.9359 (18)	174.8 (19)
N2—H2*N*2⋯O4^ii^	0.81 (2)	2.26 (2)	2.9847 (17)	149.2 (18)
O2—H1*O*2⋯O6^iii^	0.81 (2)	1.92 (2)	2.7215 (15)	170 (2)
O3—H1*O*3⋯O7^iv^	0.78 (2)	1.93 (2)	2.6988 (15)	168 (2)
O4—H1*O*4⋯O2^v^	0.80 (2)	1.97 (2)	2.7621 (15)	171 (2)
O5—H1*O*5⋯O3^vi^	0.81 (2)	1.92 (2)	2.7237 (15)	170 (2)
O6—H1*O*6⋯O1^v^	0.79 (2)	2.02 (2)	2.7548 (15)	156 (2)
O7—H1*O*7⋯N1	0.82 (1)	1.79 (2)	2.6038 (16)	175 (2)

Symmetry codes: (i) 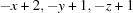; (ii) 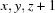; (iii) 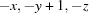; (iv) 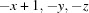; (v) 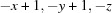; (vi) 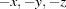.

**Table 3:** Experimental details

Crystal data	
Chemical formula	C_6_H_6_N_2_O·H_6_O_6_Te
*M*_r_	351.8
Crystal system, space group	Triclinic, *P* 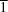
Temperature (K)	120
*a*, *b*, *c* (Å)	7.0094 (3), 7.5750 (3), 10.6149 (5)
α, β, γ (°)	70.945 (4), 78.748 (4), 89.901 (4)
*V* (Å^3^)	521.32 (4)
*Z*	2
Radiation type	Mo *K*α
μ (mm^−1^)	2.88
Crystal size (mm)	0.31 × 0.21 × 0.09

Data collection	
Diffractometer	Rigaku Oxford Diffraction Xcalibur, AtlasS2, Gemini ultra
Absorption correction	Analytical *CrysAlis PRO* (Rigaku OD, 2018 ▸)
*T*_min_, *T*_max_	0.618, 0.816
No. of measured, independent and observed [*I* > 2σ(*I*)] reflections	6995, 2408, 2254
*R*_int_	0.019
(sin θ/λ)_max_ (Å^−1^)	0.671

Refinement	
*R*[*F* ^2^ > 2σ(*F* ^2^)], *wR*(*F* ^2^), *S*	0.012, 0.032, 1.08
No. of reflections	2408
No. of parameters	170
No. of restraints	6
H-atom treatment	H atoms treated by a mixture of independent and constrained refinement
Δρ_max_, Δρ_min_ (e Å^−3^)	0.42, −0.35

Computer programs: *CrysAlis PRO* (Rigaku OD, 2018 ▸), *SIR2014* (Burla *et al.*, 2015 ▸), *SHELXL* (Sheldrick, 2015 ▸), *PLATON* (Spek, 2009 ▸) and *DIAMOND* (Brandenburg & Putz, 2005 ▸).

## supplementary crystallographic information

### Crystal data

**Table:**

C_6_H_6_N_2_O·H_6_O_6_Te	*Z* = 2
*M**_r_* = 351.8	*F*(000) = 340
Triclinic, *P*1	*D*_x_ = 2.241 Mg m^−^^3^
Hall symbol: -P 1	Mo *K*α radiation, λ = 0.71073 Å
*a* = 7.0094 (3) Å	Cell parameters from 5013 reflections
*b* = 7.5750 (3) Å	θ = 3.3–28.4°
*c* = 10.6149 (5) Å	µ = 2.88 mm^−^^1^
α = 70.945 (4)°	*T* = 120 K
β = 78.748 (4)°	Prism, colourless
γ = 89.901 (4)°	0.31 × 0.21 × 0.09 mm
*V* = 521.32 (4) Å^3^	

### Data collection

**Table:**

Rigaku Oxford Diffraction Xcalibur, AtlasS2, Gemini ultra diffractometer	2408 independent reflections
Radiation source: X-ray tube	2254 reflections with *I* > 2σ(*I*)
Graphite monochromator	*R*_int_ = 0.019
Detector resolution: 10.3567 pixels mm^-1^	θ_max_ = 28.5°, θ_min_ = 2.9°
ω scans	*h* = −9→8
Absorption correction: analytical CrysAlisPro (Rigaku OD, 2018)	*k* = −10→9
*T*_min_ = 0.618, *T*_max_ = 0.816	*l* = −14→13
6995 measured reflections	

### Refinement

**Table:**

Refinement on *F*^2^	24 constraints
Least-squares matrix: full	H atoms treated by a mixture of independent and constrained refinement
*R*[*F*^2^ > 2σ(*F*^2^)] = 0.012	*w* = 1/[σ^2^(*F*_o_^2^) + (0.0117*P*)^2^ + 0.2806*P*] where *P* = (*F*_o_^2^ + 2*F*_c_^2^)/3
*wR*(*F*^2^) = 0.032	(Δ/σ)_max_ = 0.003
*S* = 1.08	Δρ_max_ = 0.42 e Å^−^^3^
2408 reflections	Δρ_min_ = −0.35 e Å^−^^3^
170 parameters	Extinction correction: SHELXL2014 (Sheldrick, 2015), Fc^*^=kFc[1+0.001xFc^2^λ^3^/sin(2θ)]^-1/4^
6 restraints	Extinction coefficient: 0.0185 (6)

### Special details

**Table:**

Refinement. The unrestrained refinement of the H atoms of the OH groups of the telluric acid resulted in too short O—H distances: O2 H1O2 0.70 (2) Å, O3 H1O3 0.66 (2) Å, O4 H1O4 0.70 (2) Å, O5 H1O5 0.72 (2) Å, O6 H1O6 0.70 (2) Å, O7 H1O7 0.75 (2) Å.Therefore the restrained refinement has been applied. The O—H distances were retrained as 0.84 Å with elasticity 0.02 Å.

### Fractional atomic coordinates and isotropic or equivalent isotropic displacement parameters (Å^2^)

**Table:**

	*x*	*y*	*z*	*U*_iso_*/*U*_eq_	
O1	0.87715 (15)	0.43054 (16)	0.38915 (10)	0.0154 (2)	
N1	0.39465 (19)	0.18273 (18)	0.33548 (12)	0.0137 (3)	
N2	0.7726 (2)	0.3530 (2)	0.61605 (13)	0.0159 (3)	
H1N2	0.872 (3)	0.409 (3)	0.6196 (19)	0.019*	
H2N2	0.687 (3)	0.314 (3)	0.683 (2)	0.019*	
C1	0.5627 (2)	0.2754 (2)	0.48208 (14)	0.0115 (3)	
C2	0.5568 (2)	0.2446 (2)	0.36022 (14)	0.0125 (3)	
H1C2	0.6729	0.2686	0.2922	0.015*	
C3	0.2306 (2)	0.1498 (2)	0.43111 (16)	0.0147 (3)	
H1C3	0.1135	0.1124	0.4115	0.018*	
C4	0.2249 (2)	0.1682 (2)	0.55750 (15)	0.0148 (3)	
H1C4	0.1080	0.1379	0.6250	0.018*	
C5	0.3941 (2)	0.2320 (2)	0.58308 (15)	0.0132 (3)	
H1C5	0.3945	0.2459	0.6688	0.016*	
C6	0.7492 (2)	0.3584 (2)	0.49361 (15)	0.0121 (3)	
Te1	0.24981 (2)	0.25172 (2)	−0.00312 (2)	0.00795 (5)	
O2	0.26677 (16)	0.45520 (15)	0.06561 (11)	0.0126 (2)	
H1O2	0.164 (2)	0.503 (3)	0.072 (2)	0.019*	
O3	0.24036 (16)	0.05185 (15)	−0.07618 (11)	0.0118 (2)	
H1O3	0.335 (2)	−0.002 (3)	−0.073 (2)	0.018*	
O4	0.46392 (15)	0.36909 (16)	−0.15123 (11)	0.0122 (2)	
H1O4	0.533 (3)	0.429 (3)	−0.127 (2)	0.018*	
O5	0.02924 (16)	0.14607 (16)	0.13902 (11)	0.0135 (2)	
H1O5	−0.040 (3)	0.081 (3)	0.117 (2)	0.020*	
O6	0.07017 (15)	0.37675 (16)	−0.11412 (11)	0.0122 (2)	
H1O6	0.119 (3)	0.429 (3)	−0.1913 (15)	0.018*	
O7	0.42559 (16)	0.12206 (16)	0.10483 (11)	0.0131 (2)	
H1O7	0.409 (3)	0.141 (3)	0.1777 (16)	0.020*	

### Atomic displacement parameters (Å^2^)

**Table:**

	*U*^11^	*U*^22^	*U*^33^	*U*^12^	*U*^13^	*U*^23^
O1	0.0120 (5)	0.0219 (6)	0.0120 (5)	−0.0006 (4)	−0.0017 (4)	−0.0055 (4)
N1	0.0173 (7)	0.0122 (6)	0.0135 (6)	0.0022 (5)	−0.0058 (5)	−0.0053 (5)
N2	0.0112 (6)	0.0252 (7)	0.0113 (6)	−0.0033 (5)	−0.0013 (5)	−0.0066 (5)
C1	0.0125 (7)	0.0114 (7)	0.0113 (6)	0.0030 (5)	−0.0046 (5)	−0.0035 (5)
C2	0.0135 (7)	0.0121 (7)	0.0117 (7)	0.0019 (5)	−0.0022 (5)	−0.0039 (6)
C3	0.0141 (7)	0.0126 (7)	0.0185 (7)	0.0014 (6)	−0.0058 (6)	−0.0052 (6)
C4	0.0126 (7)	0.0145 (7)	0.0159 (7)	0.0015 (6)	−0.0014 (6)	−0.0040 (6)
C5	0.0161 (7)	0.0133 (7)	0.0113 (7)	0.0028 (6)	−0.0038 (6)	−0.0051 (6)
C6	0.0116 (7)	0.0130 (7)	0.0127 (7)	0.0040 (5)	−0.0038 (5)	−0.0052 (6)
Te1	0.00632 (6)	0.01029 (6)	0.00892 (6)	0.00042 (3)	−0.00173 (4)	−0.00533 (4)
O2	0.0086 (5)	0.0145 (5)	0.0193 (5)	0.0023 (4)	−0.0034 (4)	−0.0117 (4)
O3	0.0098 (5)	0.0137 (5)	0.0164 (5)	0.0022 (4)	−0.0040 (4)	−0.0100 (4)
O4	0.0081 (5)	0.0171 (6)	0.0124 (5)	−0.0035 (4)	0.0006 (4)	−0.0079 (4)
O5	0.0104 (5)	0.0189 (6)	0.0117 (5)	−0.0047 (4)	0.0006 (4)	−0.0075 (4)
O6	0.0085 (5)	0.0168 (5)	0.0106 (5)	0.0021 (4)	−0.0023 (4)	−0.0036 (4)
O7	0.0132 (5)	0.0181 (6)	0.0134 (5)	0.0066 (4)	−0.0071 (4)	−0.0100 (4)

### Geometric parameters (Å, º)

**Table:**

O1—C6	1.2450 (18)	C5—H1C5	0.9500
N1—C2	1.3337 (19)	Te1—O7	1.8994 (11)
N1—C3	1.340 (2)	Te1—O5	1.9007 (11)
N2—C6	1.3290 (19)	Te1—O3	1.9197 (10)
N2—H1N2	0.83 (2)	Te1—O4	1.9198 (11)
N2—H2N2	0.81 (2)	Te1—O2	1.9212 (10)
C1—C5	1.390 (2)	Te1—O6	1.9279 (11)
C1—C2	1.3955 (19)	O2—H1O2	0.806 (15)
C1—C6	1.495 (2)	O3—H1O3	0.781 (15)
C2—H1C2	0.9500	O4—H1O4	0.796 (15)
C3—C4	1.387 (2)	O5—H1O5	0.812 (15)
C3—H1C3	0.9500	O6—H1O6	0.789 (15)
C4—C5	1.388 (2)	O7—H1O7	0.818 (14)
C4—H1C4	0.9500		
			
C2—N1—C3	118.72 (13)	O7—Te1—O5	92.47 (5)
C6—N2—H1N2	117.2 (13)	O7—Te1—O3	90.08 (5)
C6—N2—H2N2	122.0 (14)	O5—Te1—O3	92.84 (5)
H1N2—N2—H2N2	120.0 (18)	O7—Te1—O4	90.50 (5)
C5—C1—C2	118.11 (14)	O5—Te1—O4	176.85 (4)
C5—C1—C6	124.29 (13)	O3—Te1—O4	88.21 (5)
C2—C1—C6	117.58 (13)	O7—Te1—O2	89.74 (5)
N1—C2—C1	122.63 (14)	O5—Te1—O2	89.02 (5)
N1—C2—H1C2	118.7	O3—Te1—O2	178.14 (4)
C1—C2—H1C2	118.7	O4—Te1—O2	89.94 (5)
N1—C3—C4	122.59 (14)	O7—Te1—O6	178.29 (4)
N1—C3—H1C3	118.7	O5—Te1—O6	87.35 (5)
C4—C3—H1C3	118.7	O3—Te1—O6	88.22 (5)
C3—C4—C5	118.47 (14)	O4—Te1—O6	89.72 (5)
C3—C4—H1C4	120.8	O2—Te1—O6	91.96 (5)
C5—C4—H1C4	120.8	Te1—O2—H1O2	110.5 (15)
C4—C5—C1	119.35 (13)	Te1—O3—H1O3	110.6 (15)
C4—C5—H1C5	120.3	Te1—O4—H1O4	109.1 (15)
C1—C5—H1C5	120.3	Te1—O5—H1O5	110.4 (15)
O1—C6—N2	122.04 (14)	Te1—O6—H1O6	114.5 (15)
O1—C6—C1	119.44 (13)	Te1—O7—H1O7	111.3 (14)
N2—C6—C1	118.52 (13)		
			
C3—N1—C2—C1	0.4 (2)	C2—C1—C5—C4	−2.8 (2)
C5—C1—C2—N1	2.7 (2)	C6—C1—C5—C4	175.27 (14)
C6—C1—C2—N1	−175.51 (13)	C5—C1—C6—O1	−164.04 (14)
C2—N1—C3—C4	−3.4 (2)	C2—C1—C6—O1	14.1 (2)
N1—C3—C4—C5	3.2 (2)	C5—C1—C6—N2	15.8 (2)
C3—C4—C5—C1	0.0 (2)	C2—C1—C6—N2	−166.14 (14)

### Hydrogen-bond geometry (Å, º)

**Table:**

*D*—H···*A*	*D*—H	H···*A*	*D*···*A*	*D*—H···*A*
N2—H1*N*2···O1^i^	0.83 (2)	2.11 (2)	2.9359 (18)	174.8 (19)
N2—H2*N*2···O4^ii^	0.81 (2)	2.26 (2)	2.9847 (17)	149.2 (18)
O2—H1*O*2···O6^iii^	0.81 (2)	1.92 (2)	2.7215 (15)	170 (2)
O3—H1*O*3···O7^iv^	0.78 (2)	1.93 (2)	2.6988 (15)	168 (2)
O4—H1*O*4···O2^v^	0.80 (2)	1.97 (2)	2.7621 (15)	171 (2)
O5—H1*O*5···O3^vi^	0.81 (2)	1.92 (2)	2.7237 (15)	170 (2)
O6—H1*O*6···O1^v^	0.79 (2)	2.02 (2)	2.7548 (15)	156 (2)
O7—H1*O*7···N1	0.82 (1)	1.79 (2)	2.6038 (16)	175 (2)

Symmetry codes: (i) −*x*+2, −*y*+1, −*z*+1; (ii) *x*, *y*, *z*+1; (iii) −*x*, −*y*+1, −*z*; (iv) −*x*+1, −*y*, −*z*; (v) −*x*+1, −*y*+1, −*z*; (vi) −*x*, −*y*, −*z*.
